# Supplementary material for: Audible pain squeaks can mediate emotional contagion across pre-exposed rats with a potential effect of auto-conditioning
Source: Commun Biol. 2023 Oct 25;6:1085. doi: 10.1038/s42003-023-05474-x (PMC10600148; doi:10.1038/s42003-023-05474-x)
Supplement: Supplementary file 2 — Supplementary Information [file 42003_2023_5474_MOESM2_ESM.pdf]

Supplementary Information

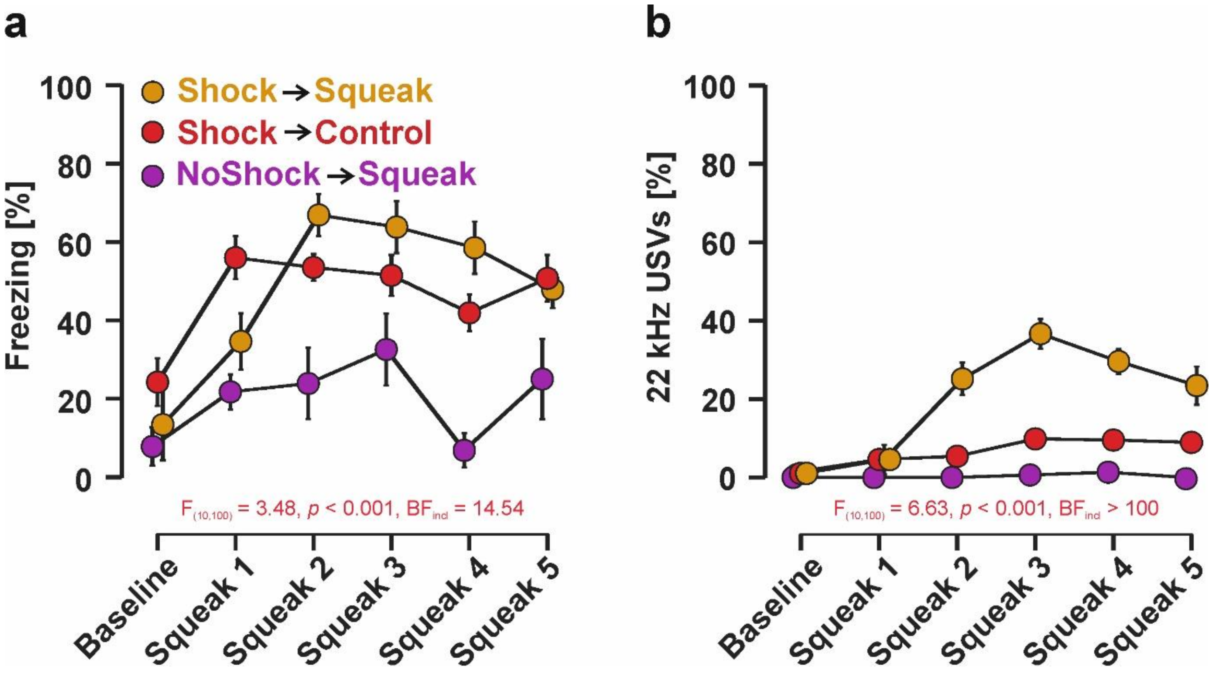

Supplementary Figure 1. Fear response rates resolved stimulus-by-stimulus over the baseline and playback period for the experimental groups of Experiment 1. a) Freezing and b) 22 kHz vocalizations were quantified over the course of each ITI in between stimulus presentations. Statistics below the graphs reflect the interaction effects between the factors Group and Time (baseline and each ITI between squeak playbacks). Error bars represent the standard error of the mean.

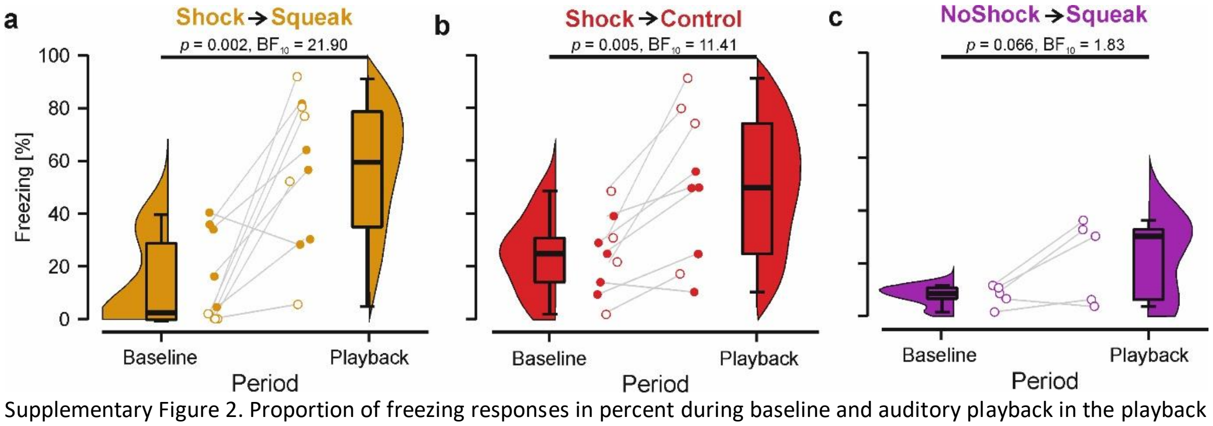

Supplementary Figure 2. Proportion of freezing responses in percent during baseline and auditory playback in the playback session. Paired tests (t-test, if the Shapiro-Wilk (S-W) normality test did not reject normality, i.e. S-W p-value>0.05 or Wilcoxon signed-rank, if it did, i.e. S-W p<0.05) indicated that both the a) Shock->Squeak group (S-W p-value = 0.773; t(9) = 4.26, p = 0.002, BF_{10} = 21.90) and the b) Shock->Control group (S-W p-value = 0.666; t(8) = 3.86, p = 0.005, BF_{10} 11.41) increased their freezing from baseline to the playback period whereas the c) NoShock->Squeak group showed a trend towards a significant increase (S-W p-value = 0.207; t(4) = 2.52, p = 0.066, BF_{10} = 1.83). Animals with higher amplitude playback are marked by an open circle. The p-values indicated for the changes from baseline to playback were not corrected for multiple comparisons, but applying an FDR correction would not alter the conclusion. Boxplots reflect the median, the first and third quartile and the interquartile range.

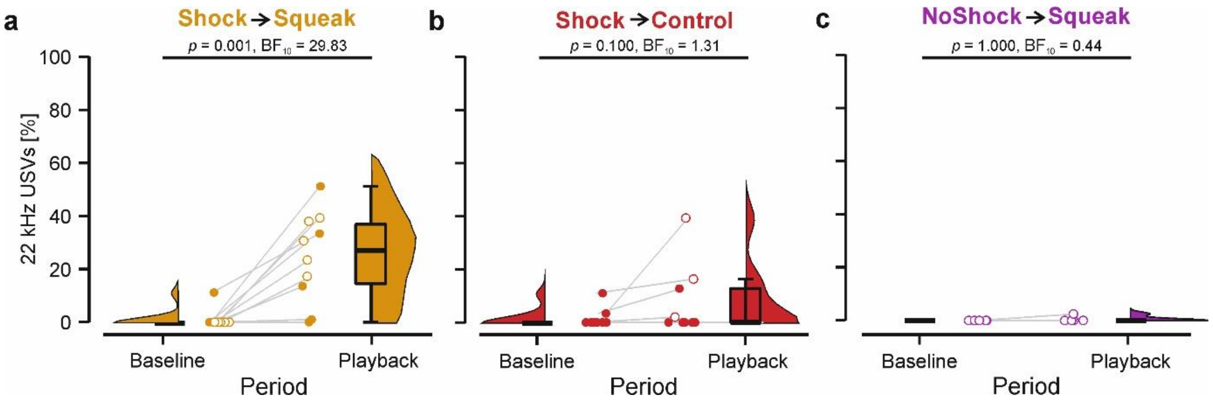

Supplementary Figure 3. Proportion of 22 kHz calls in percent during baseline and auditory playback in the playback session. For 22 kHz calls, only the a) Shock->Squeak group showed an increase from baseline to playback (S-W p-value = 0.852; t(9) = 4.51, p = 0.001, BF_{10} = 29.83). The b) Shock->Control (S-W p-value < 0.001; z = 1.83, p = 0.100, BF_{10} = 1.31) and c) NoShock->Squeak (S-W p-value < 0.001; z = 0.48, p = 1.000, BF_{10} = 0.44) showed no significant change. Animals with higher amplitude playback are marked by an open circle. The p-values indicated for the changes from baseline to playback were not corrected for multiple comparisons, but applying an FDR correction would not alter the conclusion. Boxplots reflect the median, the first and third quartile and the interquartile range.

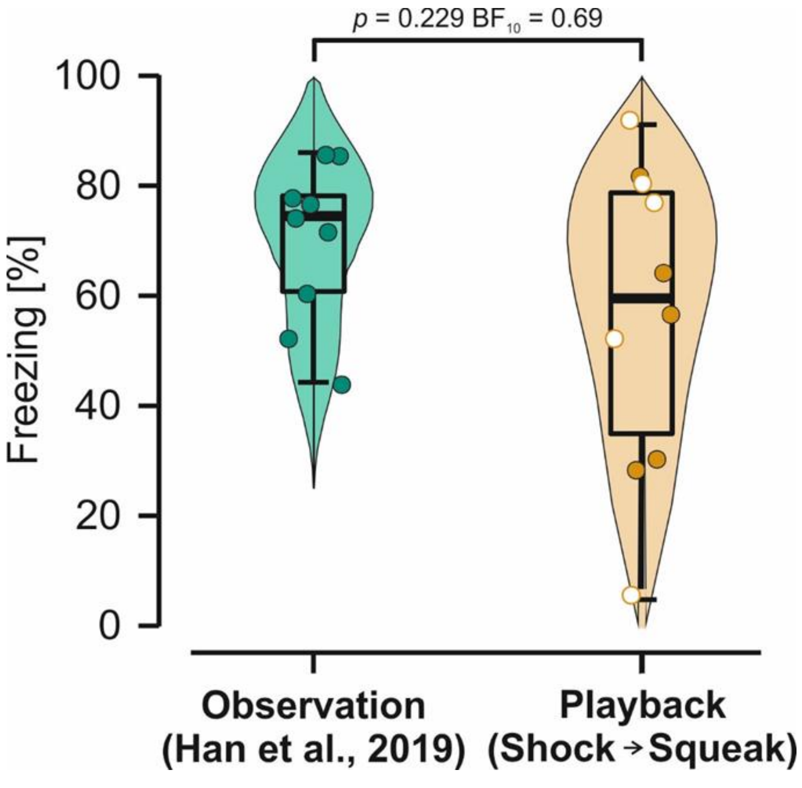

Supplementary Figure 4. Proportion of freezing responses comparing the results from Han et al. (2019) that used a real-life demonstrator during emotional contagion vs. our Shock -> Squeak group that merely played back the squeak. Animals with higher amplitude playback are marked by an open circle. Boxplots reflect the median, the first and third quartile and the interquartile range.

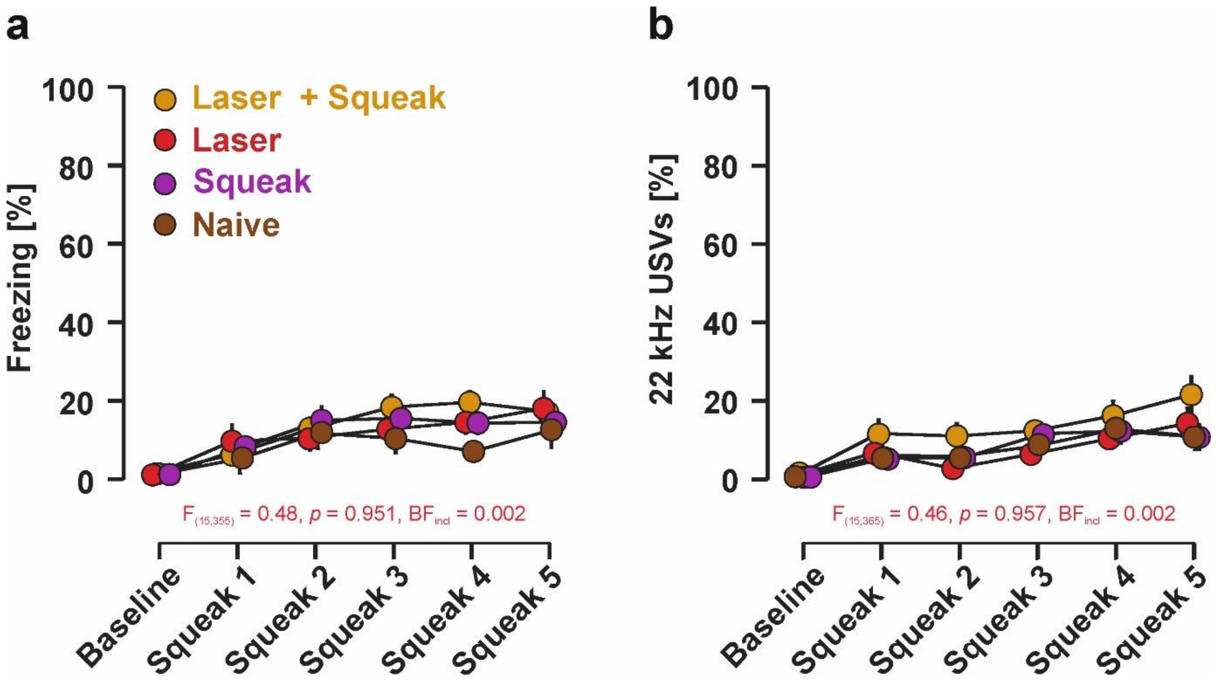

Supplementary Figure 5. Fear response rates resolved stimulus-by-stimulus over the baseline and playback period for the experimental groups of Experiment 2. a) Freezing and b) 22 kHz vocalizations were quantified over the course of each ITI in between stimulus presentations. Statistics below the graphs reflect the interaction effects between the factors Group and Time (baseline and each ITI between squeak playbacks). Error bars represent the standard error of the mean.

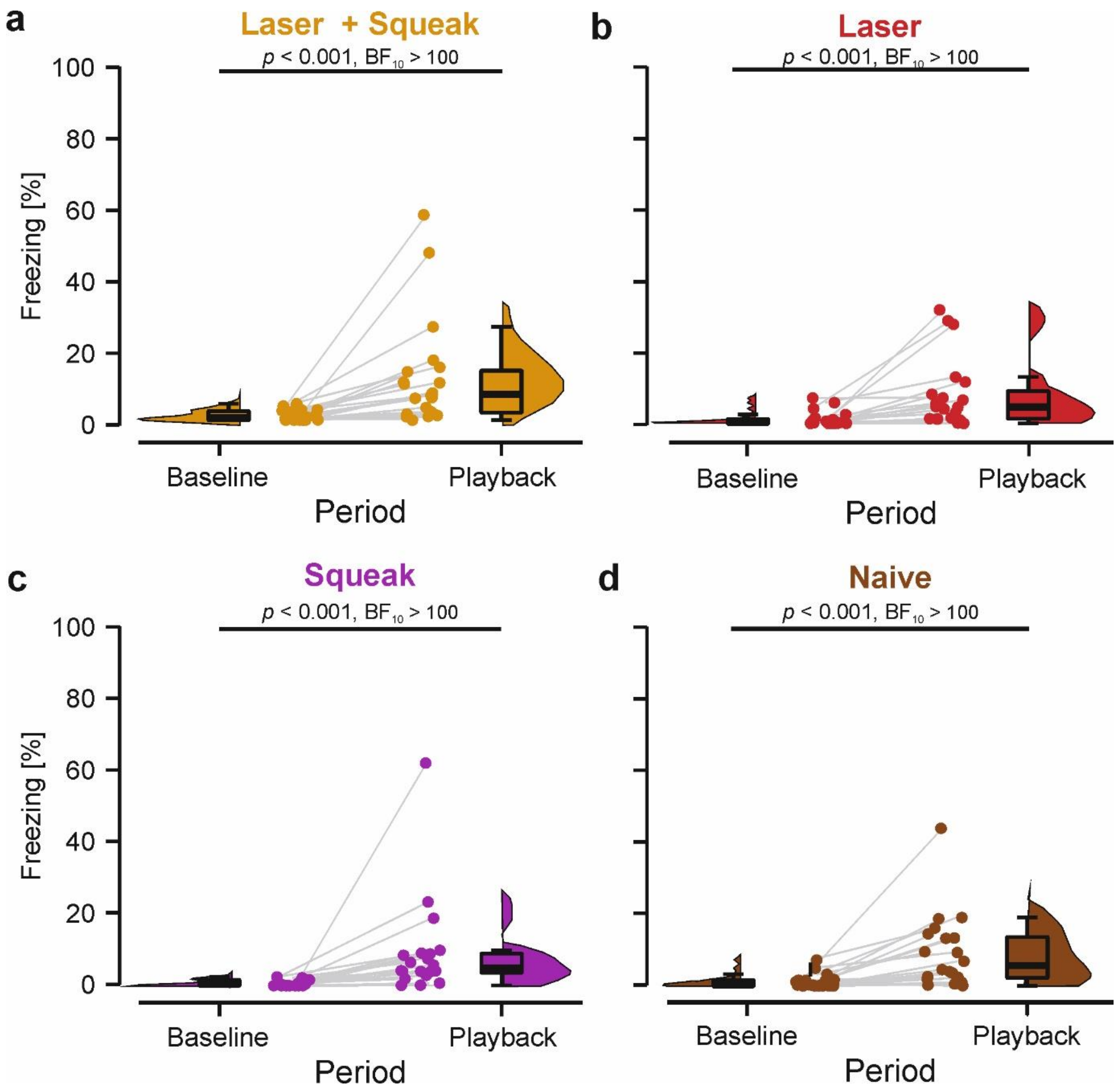

Supplementary Figure 6. Proportion of freezing responses in percent during the squeak playback session. Paired Wilcoxon signed-rank tests indicated extreme evidence for the a) Laser + Squeak group (S-W p-value < 0.001; z = 3.82, p < 0.001, BF_{10} > 100), the b) Laser group S-W p-value < 0.001; z = 3.81, p < 0.001, BF_{10} > 100), the c) Squeak group S-W p-value < 0.001; z = 3.92, p < 0.001, BF_{10} > 100), and the d) Naïve group (S-W p-value < 0.001; z = 3.61, p < 0.001, BF_{10} > 100) for an increase in freezing from the baseline to the playback period. The p-values indicated for the changes from baseline to playback were not corrected for multiple comparisons, but applying an FDR correction would not alter the conclusion. Boxplots reflect the median, the first and third quartile and the interquartile range.

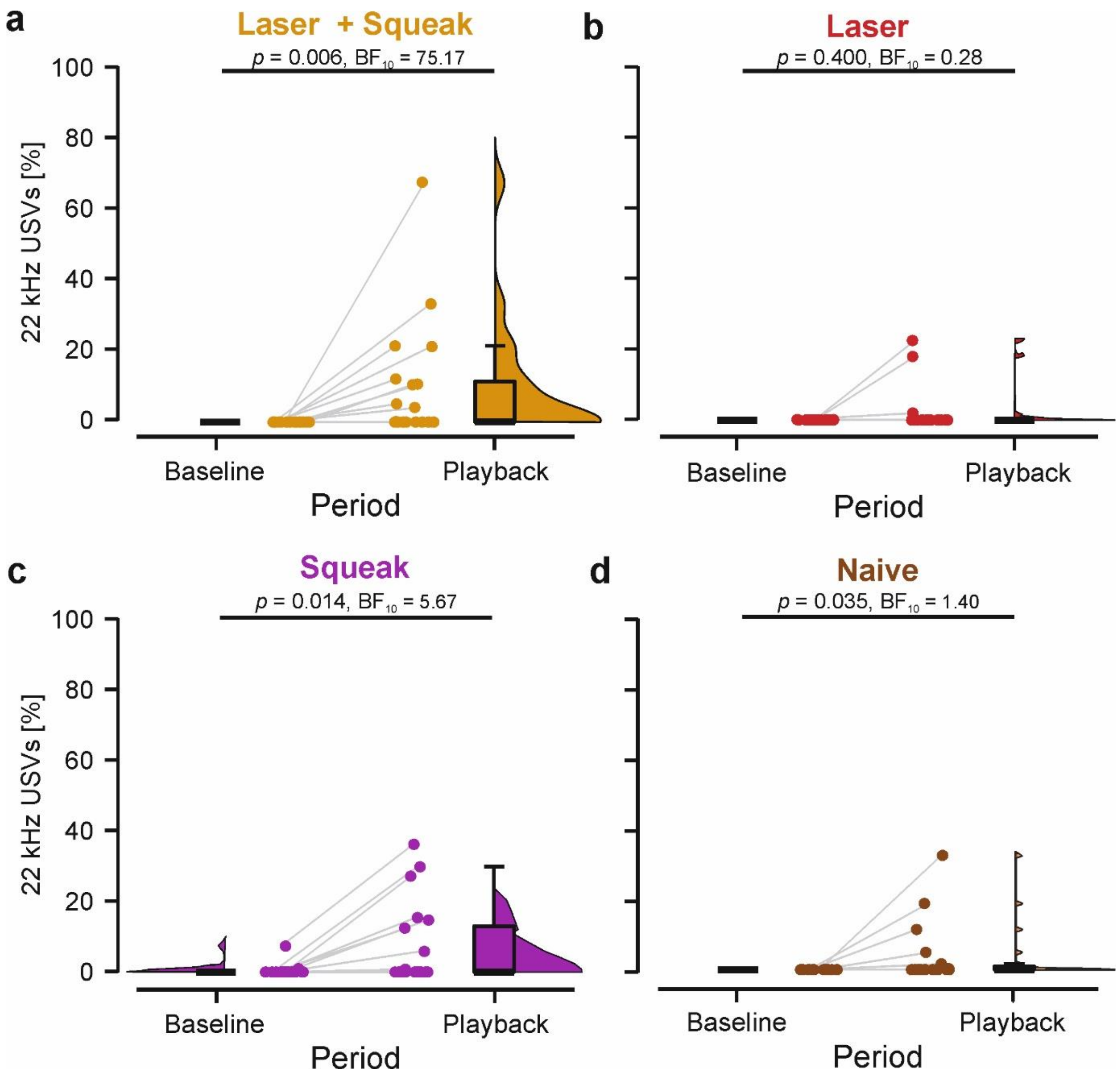

Supplementary Figure 7. Proportion of 22 kHz calls in percent during the squeak playback session. The a) Laser+Squeak group demonstrated strong to extreme evidence for an increase from the baseline to the playback period (S-W p-value < 0.001; z = 2.80, p = 0.006, BF_{10} = 75.17) whereas moderate evidence of absence of this effect was observed for the b) Laser group (S-W p-value < 0.001; z = 0.94, p = 0.400, BF_{10} = 0.28). For the c) Squeak group, we found moderate evidence in favor of an increase in 22 kHz calls (S-W p-value < 0.001; z = 2.52, p = 0.014, BF_{10} = 5.67). No evidence in either direction was found for the d) Naïve group (S-W p-value < 0.001; z = 2.20, p = 0.035, BF_{10} = 1.40). The p-values indicated for the changes from baseline to playback were not corrected for multiple comparisons, but applying an FDR correction would not alter the conclusion. Boxplots reflect the median, the first and third quartile and the interquartile range.

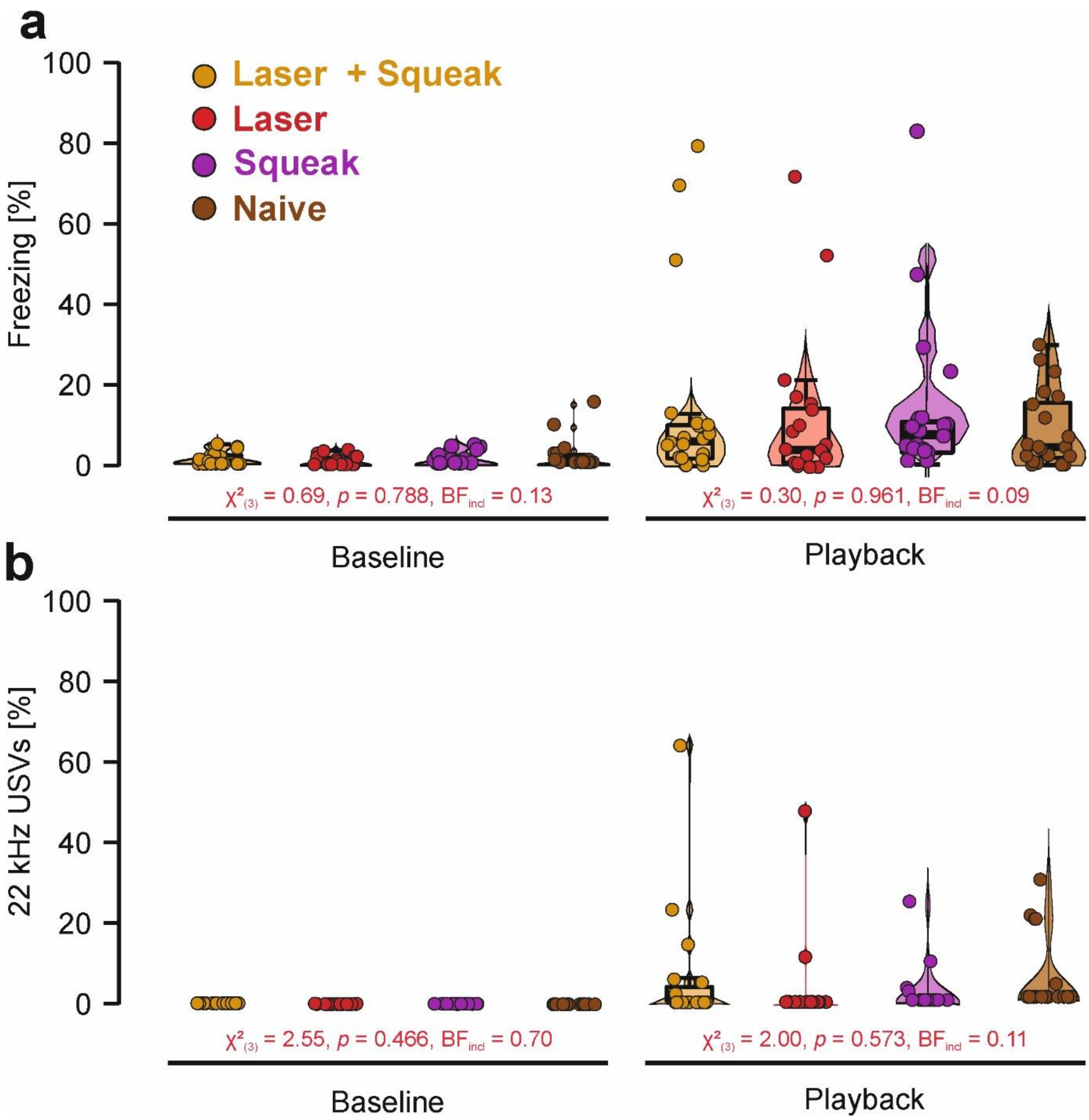

Supplementary Figure 8. Behavioral responses to the scrambled squeak playback. a) Proportion of freezing responses in percent during baseline and auditory playback in the playback session on day 7. Animals with higher amplitude playback are marked by an open circle. Only between group differences are presented here. Within group differences from baseline to playback are depicted in Supplementary Figure 9. b) Proportion of 22 kHz vocalizations in percent during baseline and auditory playback in the playback session on day 7. Animals with higher amplitude playback are marked by an open circle. Only between group differences are presented here. Within group differences from baseline to playback are depicted in Supplementary Figure 10. Boxplots reflect the median, the first and third quartile and the interquartile range.

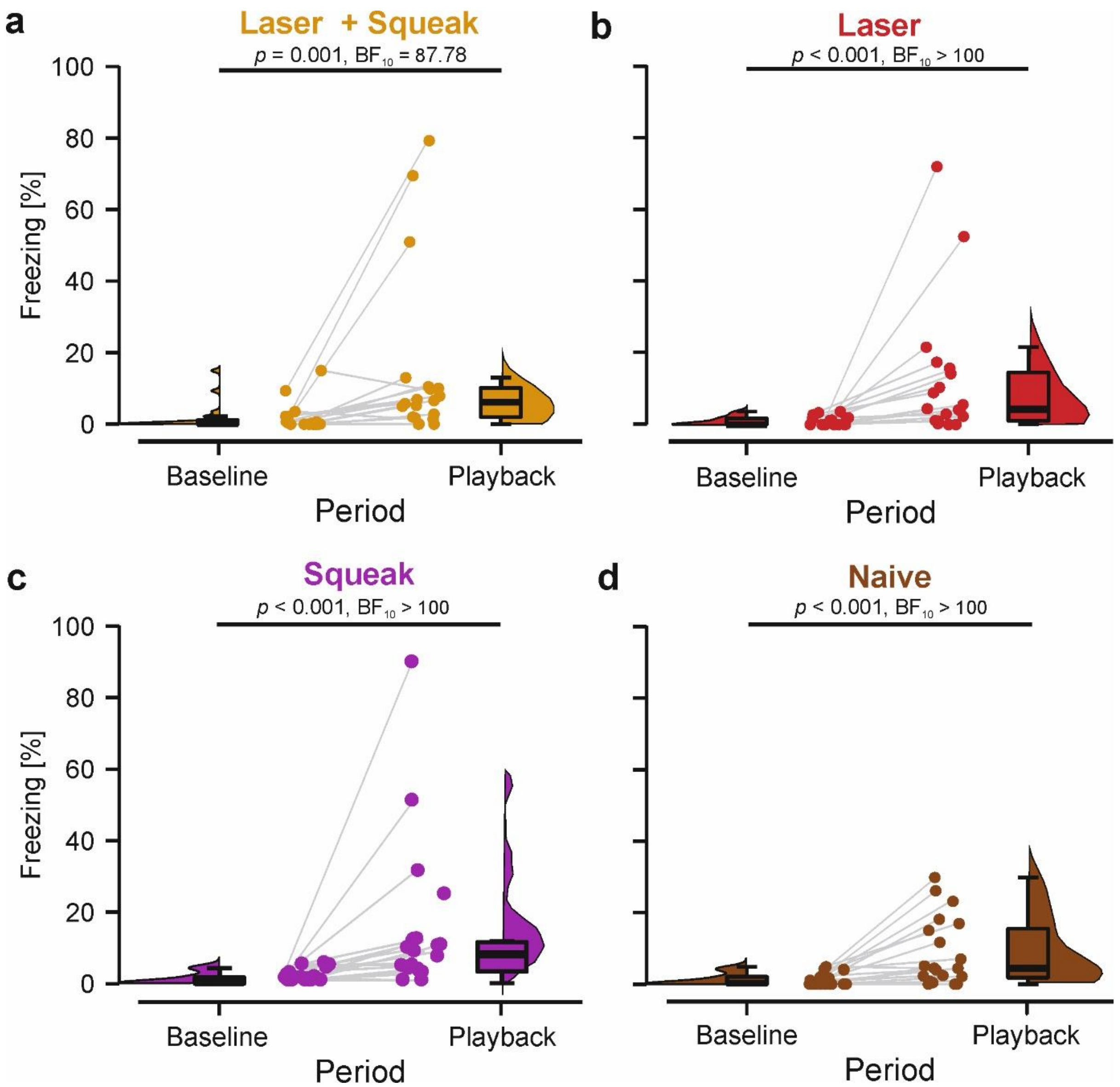

Supplementary Figure 9. Proportion of freezing responses in percent during the phase-scrambled squeak playback session. Paired Wilcoxon signed-ranked tests indicated strong to extreme evidence for the a) Laser + Squeak group (S-W p-value < 0.001; z = 3.25, p = 0.001, $BF_{10} > 87.78$), the b) Laser group S-W p-value < 0.001; z = 3.64, p < 0.001, $BF_{10} > 100$), the c) Squeak group S-W p-value < 0.001; z = 3.66, p < 0.001, $BF_{10} > 100$), and the d) Naïve group (S-W p-value < 0.001; z = 3.57, p < 0.001, $BF_{10} > 100$) for an increase in freezing from the baseline to the playback period. The p-values indicated for the changes from baseline to playback were not corrected for multiple comparisons, but applying an FDR correction would not alter the conclusion. Boxplots reflect the median, the first and third quartile and the interquartile range.

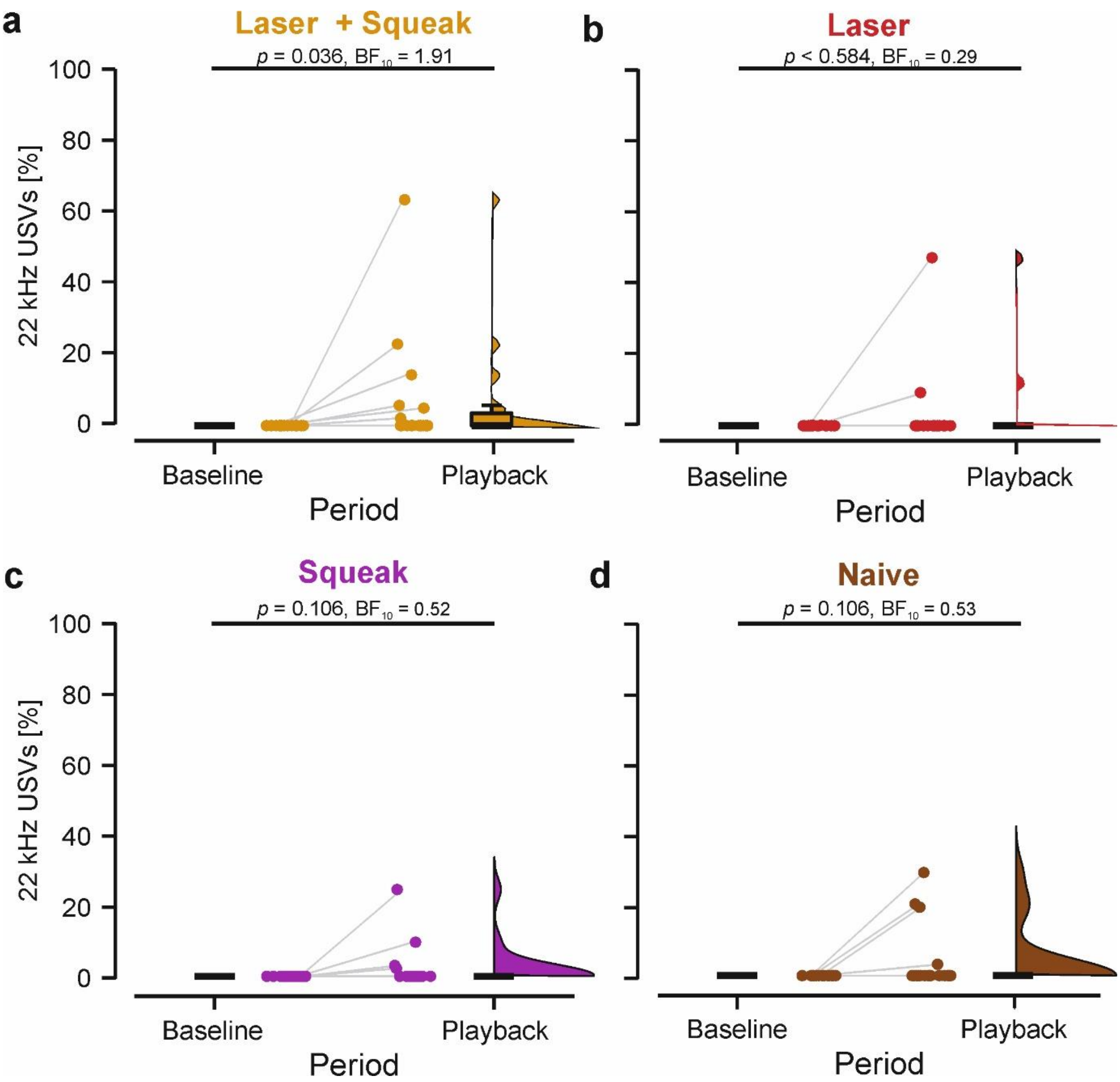

Supplementary Figure 10. Proportion of 22 kHz vocalizations in percent during the phase-scrambled squeak playback session. We found a significant increase from baseline to playback in the a) Laser+Squeak group (S-W p-value < 0.001; z = 2.20, p = 0.036).The evidence for this increase was however only anecdotal (BF_{10} = 1.91). For the b) Laser group there was moderate evidence of absence for an increase in 22 kHz calls during playback compared to baseline (S-W p-value < 0.001; z = 0.73, p = 0.584, BF_{10} = 0.29). For the c) Squeak group (S-W p-value < 0.001; z = 1.75, p = 0.106, BF_{10} = 0.52) and the d) Naïve group (S-W p-value < 0.001; z = 1.75, p = 0.106, BF_{10} = 0.53), there was absence of evidence for any effect. The p-values indicated for the changes from baseline to playback were not corrected for multiple comparisons, but applying an FDR correction would not alter the conclusion. Boxplots reflect the median, the first and third quartile and the interquartile range.

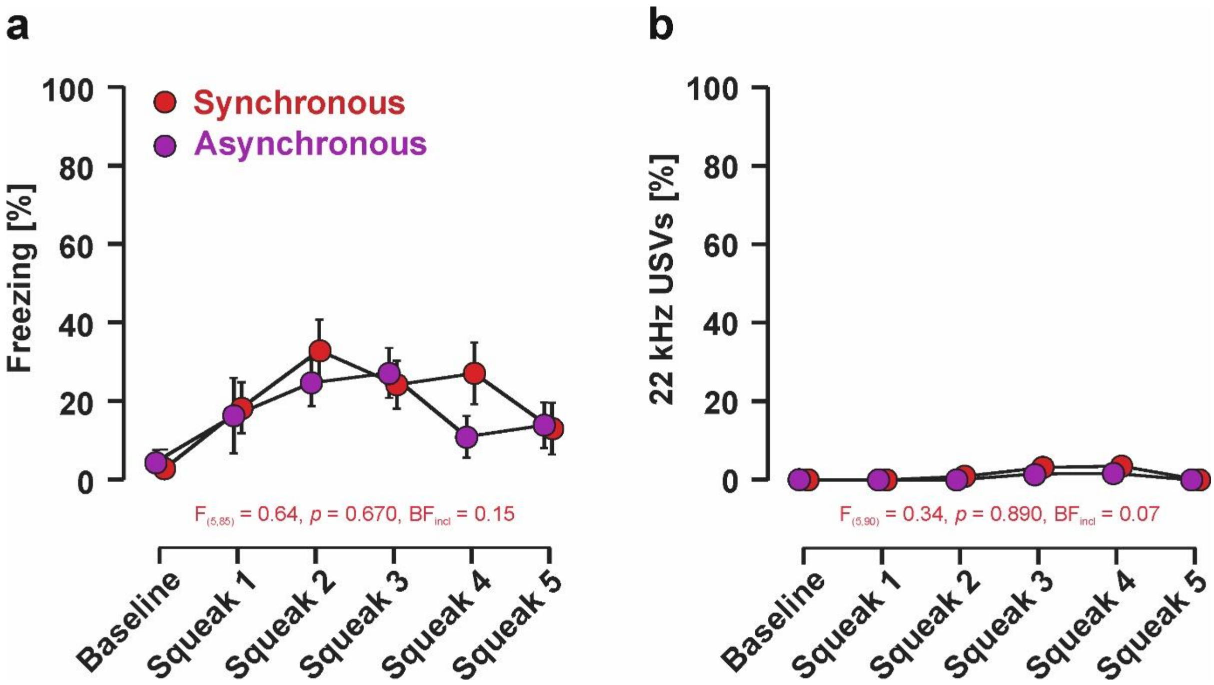

Supplementary Figure 11. Fear response rates resolved stimulus-by-stimulus over the baseline and playback period for the experimental groups of Experiment 3. a) Freezing and b) 22 kHz vocalizations were quantified over the course of each ITI in between stimulus presentations. Statistics below the graphs reflect the interaction effects between the factors Group and Time (baseline and each ITI between squeak playbacks). Error bars represent the standard error of the mean.

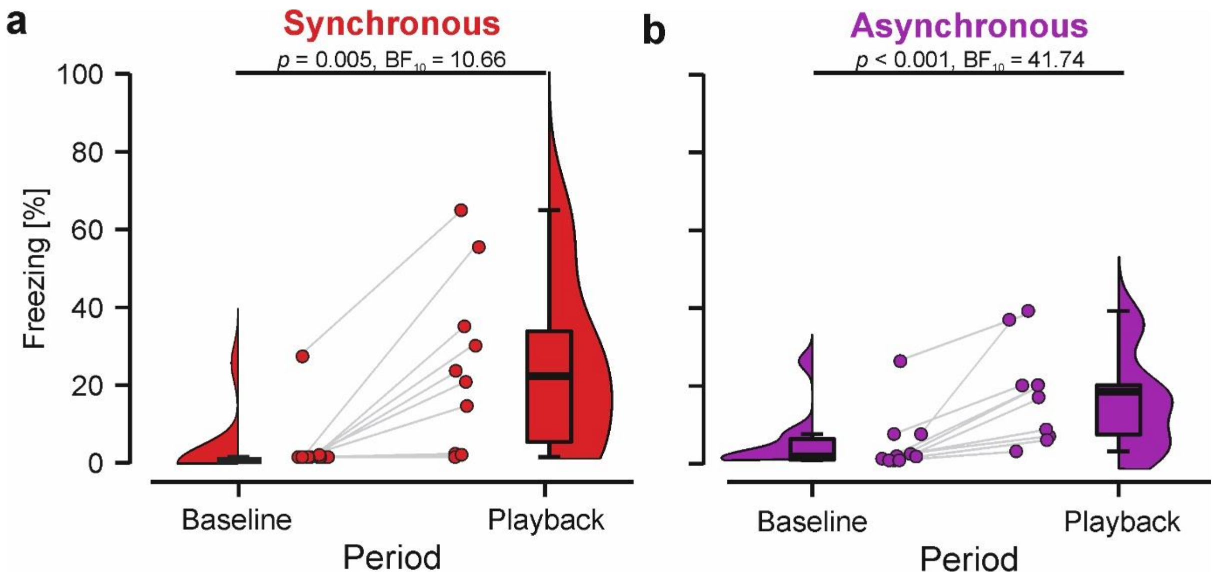

Supplementary Figure 12. Proportion of freezing responses in percent during the playback session of Experiment 3. We found a significant increase from baseline to playback in the a) Synchronous (S-W p-value = 0.502; $t_{(9)} = 3.67$, $p = 0.005$, $BF_{10} = 10.66$) and b) Asynchronous group (S-W p-value = 0.558; $t_{(9)} = 4.80$, $p < 0.001$, $BF_{10} = 41.74$). The p-values indicated for the changes from baseline to playback were not corrected for multiple comparisons, but applying an FDR correction would not alter the conclusion. Boxplots reflect the median, the first and third quartile and the interquartile range.

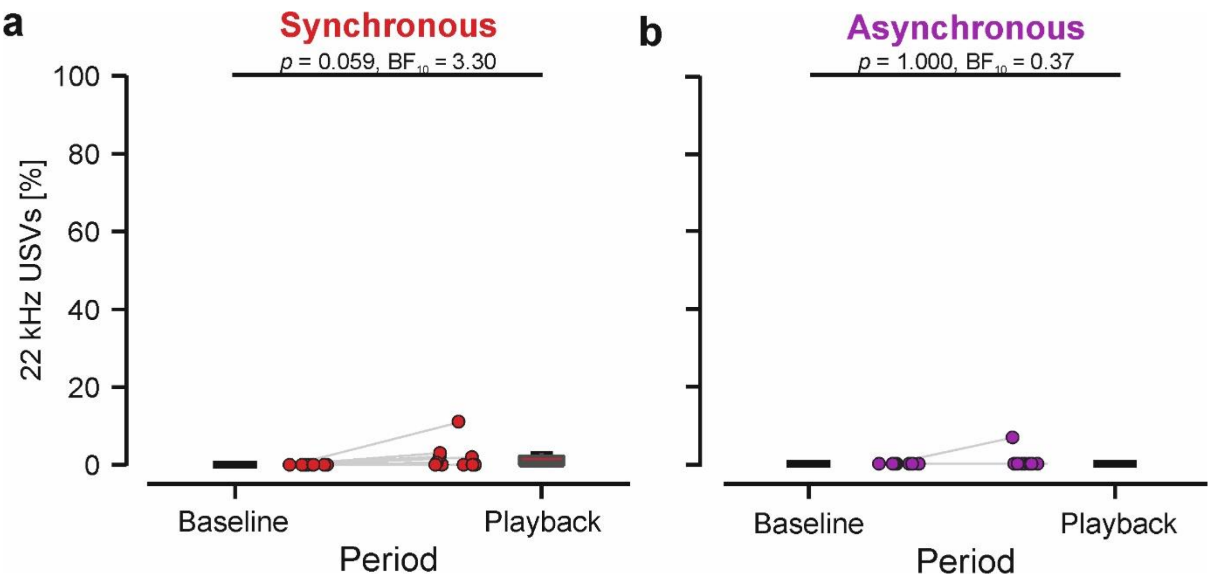

Supplementary Figure 13. Proportion of 22 kHz vocalizations in percent during the playback session of Experiment 3. We found a trend towards a significant increase from baseline to playback in the a) Synchronous (S-W p-value < 0.001; z = 2.02, p = 0.059, BF_{10} = 3.30), but no change in the b) Asynchronous group (S-W p-value < 0.001; z = 0.48, p = 1.000, BF_{10} = 0.37). The p-values indicated for the changes from baseline to playback were not corrected for multiple comparisons, but applying an FDR correction would not alter the conclusion. Boxplots reflect the median, the first and third quartile and the interquartile range.

Supplementary Table 1. Descriptive values for the proportions of freezing during pre-exposure in Experiment 1.

**Proportion of Freezing during Pre-exposure**		
**Group**	Mean	SD
**Shock->Squeak**	56.6	5.8
**Shock->Control**	53.8	8.7
**NoShock->Squeak**	4.4	6.3

Supplementary Table 2. Descriptive values for the proportions of 22 kHz vocalizations during pre-exposure in Experiment 1.

**Proportion of Freezing during Pre-exposure**		
**Group**	Mean	SD
**Shock->Squeak**	29.7	19.3
**Shock->Control**	40.2	9.8

Supplementary Table 3. Descriptive values for the proportions of freezing for the baseline and auditory playback period across the experimental groups in Experiment 1.

**Proportion of Freezing during Auditory Playback**			
**Period**	Group	Mean	SD
	Shock->Squeak	13.4	16.8
**Baseline**	Shock->Control	24.2	14.6
	NoShock->Squeak	7.8	4
	Shock->Squeak	56.7	28
**Playback**	Shock->Control	50.3	28.6
	NoShock->Squeak	21.9	15.6

Supplementary Table 4. Descriptive values for the proportions of 22 kHz calls for the baseline and auditory playback period across the experimental groups in Experiment 1.

Proportion of 22 kHz Calls during Auditory Playback			
**Period**	Group	Mean	SD
	Shock->Squeak	1.1	3.5
**Baseline**	Shock->Control	1.6	3.7
	NoShock->Squeak	0	0
	Shock->Squeak	24.8	16.8
**Playback**	Shock->Control	7.8	13.4
	NoShock->Squeak	0.5	1.1

Supplementary Table 5. Descriptive values for the pain reactions to the laser during pre-exposure in Experiment 2.

Cumulative Pain Reactions during Pre-exposure		
**Group**	Mean	SD
**Laser+Squeak**	9.1	2
**Laser**	9.4	1.5
**Squeak**	2.1	1.9
**Naive**	0.9	1.1

Supplementary Table 6. Descriptive values for the proportions of freezing during pre-exposure in Experiment 2.

**Proportion of Freezing during Pre-exposure**		
**Group**	Mean	SD
**Laser+Squeak**	24.9	20.4
**Laser**	26.8	21.3
**Squeak**	26.9	20.5
**Naive**	17	12.5

Supplementary Table 7. Descriptive values for the proportions of freezing during pre-exposure in Experiment 2.

**Proportion of 22 kHz Calls during Pre-exposure**		
**Group**	Mean	SD
**Laser+Squeak**	0.8	2.6
**Laser**	0.4	1.9
**Squeak**	0	0
**Naive**	0.8	3.8

Supplementary Table 8. Descriptive values for the proportions of freezing for the baseline and auditory playback period during regular squeak playback across the experimental groups in Experiment 2.

**Proportion of Freezing during Squeak Playback**			
**Period**	Group	Mean	SD
	Laser+Squeak	1.7	2
**Baseline**	Laser	1.3	2.1
	Squeak	0.9	1.1
	Naive	1.8	2.5
	Laser+Squeak	15	17.6
**Playback**	Laser	8.6	9.6
	Squeak	10.3	13.7
	Naive	10.6	12.3

Supplementary Table 9. Descriptive values for the proportions of 22 kHz calls for the baseline and auditory playback period during regular squeak playback across the experimental groups in Experiment 2.

**Proportion of 22 kHz Calls during Squeak Playback**			
**Period**	Group	Mean	SD
	Laser+Squeak	0	0
**Baseline**	Laser	0.1	1.4
	Squeak	1.2	1.7
	Naive	0.4	1.6
	Laser+Squeak	9.9	17.1
**Playback**	Laser	2.2	6.4
	Squeak	7.1	11.6
	Naive	3.7	8.5

Supplementary Table 10. Descriptive values for the proportions of freezing for the baseline and auditory playback period during phase-scrambled squeak playback across the experimental groups in Experiment 2.

**Proportion of Freezing during Phase-Scrambled Squeak Playback**			
**Period**	Group	Mean	SD
	Laser+Squeak	2	3.8
**Baseline**	Laser	1.1	1.4
	Squeak	1.2	1.7
	Naive	1.6	2.3
	Laser+Squeak	15	23.1
**Playback**	Laser	13.2	18.7
	Squeak	14.1	20.9
	Naive	9.8	10.1

Supplementary Table 11. Descriptive values for the proportions of 22 kHz calls for the baseline and auditory playback period during phase-scrambled squeak playback across the experimental groups in Experiment 2.

**Proportion of 22 kHz Calls during Phase-Scrambled Squeak Playback**			
**Period**	Group	Mean	SD
	Laser+Squeak	0	0
**Baseline**	Laser	0.1	0.4
	Squeak	0	0
	Naive	0	0
	Laser+Squeak	6	15.2
**Playback**	Laser	3.2	11.3
	Squeak	2.2	6
	Naive	3.8	8.7

Supplementary Table 12. Descriptive values for the proportions of freezing during pre-exposure in Experiment 3.

**Proportion of Freezing during Pre-exposure**		
**Group**	Mean	SD
**Synchronous**	20.0	18.7
**Asynchronous**	13.2	17.2

Supplementary Table 13. Descriptive values for the proportions of freezing during pre-exposure in Experiment 3.

**Proportion of Freezing during Pre-exposure**		
**Group**	Mean	SD
**Synchronous**	5.2	11.0
**Asynchronous**	3.2	8.1

Supplementary Table 14. Descriptive values for the proportions of freezing for the baseline and auditory playback period during squeak playback across the experimental groups in Experiment 3.

**Proportion of Freezing during Squeak Playback**			
**Period**	Group	Mean	SD
**Baseline**	Synchronous	4.3	7.9
	Asynchronous	2.7	8.2
**Playback**	Synchronous	23.6	22.1
	Asynchronous	17.0	12.4

Supplementary Table 15. Descriptive values for the proportions of 22 kHz vocalizations for the baseline and auditory playback period during squeak playback across the experimental groups in Experiment 3.

**Proportion of 22 kHz vocalizations during Squeak Playback**			
**Period**	Group	Mean	SD
**Baseline**	Synchronous	0	0
	Asynchronous	0	0
**Playback**	Synchronous	1.9	3.4
	Asynchronous	0.7	2.1
